# Anodal Motor-Cortex Transcranial Direct Current Stimulation and Explosive Lower-Limb Power in Healthy Active Adults: A Sham-Controlled Systematic Review and Meta-Analysis

**DOI:** 10.3390/sports14090401

**Published:** 2026-09-11

**Authors:** Taeseok Choi, Hanshin Jeong, Yohan Uhm

**Affiliations:** Department of Physical Therapy, Kunjang University, Gunsan 54045, Republic of Korea; buycho@kunjang.ac.kr (T.C.); hsjung@kunjang.ac.kr (H.J.)

**Keywords:** transcranial direct current stimulation, motor cortex, vertical jump, rate of force development, explosive power, neuromodulation, meta-analysis

## Abstract

Anodal transcranial direct current stimulation (tDCS) over the primary motor cortex (M1) has been proposed as an ergogenic aid. Prior reviews pooled heterogeneous outcomes, and none isolated explosive lower-limb power or separated jump output from force–time kinetics. We asked whether anodal M1 tDCS versus sham improves explosive lower-limb power in healthy active adults, analysing these two outcome categories separately. Following PRISMA 2020 (PROSPERO CRD420261438841), four databases were searched up to 3 July 2026 for randomised and crossover trials. Random-effects meta-analyses (REML with Hartung–Knapp) pooled Hedges’ *g* by category; risk of bias (RoB 2) and certainty (GRADE) were assessed. Seven trials were included. tDCS did not significantly improve jump output (*k* = 6; *g* = 0.44, 95% CI −0.05 to 0.92; *p* = 0.069; *I*^2^ = 19%). The kinetics estimate was moderate (*k* = 3; *g* = 0.67, 95% CI 0.53 to 0.81; *I*^2^ = 0%) but did not survive a conservative small-sample correction (95% CI −0.23 to 1.57; *p* = 0.086). Certainty was very low throughout. Including a further trial whose comparator was a no-intervention control made both estimates significant, indicating a fragile evidence base. Current evidence does not establish an ergogenic benefit; adequately powered, pre-registered trials are needed.

## 1. Introduction

Explosive lower-limb power—the ability to generate high muscular force in a very short time—is a decisive, trainable determinant of competitive performance in the jumping, sprinting, and change-of-direction actions that pervade most sports [1]. Maximal neuromuscular power reflects the interaction of the force- and velocity-generating capacities of the muscle–tendon unit and is strongly shaped by neural drive [2]. Two conceptually distinct but complementary sets of measures capture it. The first is jump output: jump height or distance in the countermovement jump (CMJ), the squat jump (SJ), or the drop jump (DJ). The second is force–time kinetics, comprising the rate of force development (RFD), peak and mean power, and peak force.

These two levels are not interchangeable. Jump height is an integrated end result achievable through different force–time strategies, whereas force–time kinetics—particularly the RFD—directly index the nervous system’s capacity to rapidly recruit and drive motor units in the first milliseconds of contraction [3]. RFD and early-phase force production are highly dependent on neural drive and among the most responsive variables to changes in corticospinal excitability [4]. An intervention acting on the nervous system might modify force–time kinetics without necessarily changing integrated jump height. Synthesising these two categories separately therefore offers a more informative test than pooling them.

Transcranial direct current stimulation (tDCS) has been proposed as a non-invasive ergogenic aid for athletic performance [5,6]. A weak scalp current alters cortical excitability, and anodal stimulation over M1 increases corticospinal excitability that could translate into greater explosive output. Because anodal M1 tDCS is theorised to augment neural drive, explosive lower-limb power—and its kinetic component in particular—is a mechanistically coherent target.

Despite this rationale, the evidence remains contested. Early meta-analyses reported small pooled effects with substantial heterogeneity and dependence on few studies [7,8]. Later syntheses reported modest effects while noting jumping among the more responsive tasks [9], and a single-session synthesis in athletes found a moderate benefit that was clearest for visuomotor tasks [10]. Others have been more guarded [11]. An umbrella review of nine meta-analyses concluded that the average effect did not survive correction for publication bias [12]. Trial-level results are mixed [13], possibly reflecting heterogeneity of pooled outcomes, populations, and stimulation parameters.

No review has isolated explosive lower-limb power or distinguished jump output from force–time kinetics under sham-controlled conditions. Separating them, while restricting eligibility to healthy active adults, provides a mechanistically precise and interpretable synthesis. We hypothesised that anodal M1 tDCS would produce sham-controlled improvements in explosive lower-limb power, and that any such effect would be larger for force–time kinetics than for jump output.

## 2. Materials and Methods

### 2.1. Protocol and Registration

This review followed PRISMA 2020 [14,15] (completed checklist in Table S2) and the Cochrane Handbook, version 6.4 [16]. It was prospectively registered in PROSPERO (CRD420261438841). Deviations from the registered protocol are reported transparently in Section 4.5.

### 2.2. Eligibility Criteria

Eligibility criteria are set out in full in Table 1. In brief, we included randomised parallel-group and randomised crossover trials in healthy adults aged 18–45 years, ranging from recreationally active to trained, in which anodal tDCS was delivered over M1 and compared with sham (placebo) stimulation. Two pre-specified outcome categories were analysed separately: jump output (CMJ, countermovement jump with arm swing [CMJA], SJ, or DJ height or distance) and force–time kinetics (RFD, peak or mean power, peak force).

Comparator. The registered protocol required a genuine sham condition and listed studies without one as an explicit exclusion. Accordingly, trials whose comparator was a no-intervention or resting control were not eligible.

### 2.3. Information Sources and Search

Four databases were searched from inception to 3 July 2026 without language restriction: MEDLINE (via PubMed), Embase, Web of Science Core Collection, and Cochrane CENTRAL. The strategy combined three concept blocks with the Boolean operator AND. Terms within each block were combined with OR. Controlled vocabulary (MeSH, Emtree) was combined with title and abstract free-text terms. The blocks were as follows:

- Block 1—intervention: transcranial direct current stimulation; tDCS; anodal stimulation; anodal; brain stimulation; neuromodulation; transcranial electrical stimulation; tES; direct current stimulation
- Block 2—target: motor cortex; motor cortical; M1; primary motor; corticospinal; corticomotor
- Block 3—outcome: jump; jumping; CMJ; CMJA; countermovement; squat jump; drop jump; vertical jump; plyometric; explosive; explosive power; ballistic; muscle power; muscular power; lower-limb power; peak power; mean power; rate of force development; RFD; peak force; force–time

Restrictions on population, comparator, and design were applied at screening rather than within the search string, so as to preserve sensitivity. The complete strategy for each database, with line-by-line syntax, is given in Table S1.

The protocol pre-specified six databases. Scopus and SPORTDiscus could not be accessed through institutional subscriptions, so the final search comprised four. This deviation was mitigated by backward and forward citation tracking. We tracked all included studies and the principal prior reviews [7,8,9,10,11,12,13]. Web of Science and Scopus overlap substantially, so the omission of Scopus is unlikely to have materially affected retrieval [17].

### 2.4. Study Selection and Data Extraction

Records were de-duplicated and screened against the eligibility criteria, followed by full-text assessment. Screening, data extraction, risk-of-bias assessment, and GRADE rating were conducted primarily by a single assessor (T.C.). The protocol specified two independent reviewers; this was not achieved, and inter-rater agreement (Cohen’s kappa) is therefore not reported. To quantify the consequences of single-assessor screening, the corresponding author (Y.U.), who was not involved in the original screening, carried out a verification audit. He independently re-assessed a random 20% sample (*n* = 9) of the 45 reports sought for full-text retrieval, drawn with a fixed random seed (Python 3, random.sample, seed 20260808) so that the sample is reproducible. The sampling frame and the drawn records are available from the corresponding author on request. He also re-checked every extracted value for all included trials against the source publications.

For screening decisions, agreement was 8 of 9. The single disagreement concerned Zhou et al. [18]. The original screen had advanced that trial as eligible even though its comparator was a no-intervention control. The audit judged it ineligible, and it was removed from the primary analyses accordingly (Section 3.1 and Section 3.6). For data extraction, all 36 extracted values across the nine outcome rows matched the source reports. Two standard deviations required derivation rather than direct transcription: Lu et al. [19] reported dispersion as the standard error of the mean, and standard deviations were obtained as SE × √n, as described above.

Data were extracted on a piloted form: study characteristics, participants, stimulation parameters, comparator condition, outcome measures, and post-intervention means and standard deviations by condition. Where only standard errors or confidence intervals were reported, standard deviations were derived by standard formulae. Where a trial reported several eligible measures within one outcome category, we used the measure named in the registered protocol; peak power was therefore taken from Zhu et al. [20] rather than peak torque, which the protocol does not list. That rule did not settle the case completely. Zhu et al. report peak power separately for the ankle, knee and hip, and the protocol names peak or mean power without naming a joint. We used knee power, which is the kinetic variable that the trial reports as significant, and we give the alternatives in Section 3.4. Where several assessment times were reported, we used the time immediately after stimulation to align with the acute designs that predominate. Where a trial included more than one active condition, we used the condition the original authors designated as primary—the 4 mA condition in Chen et al. [21]—and report the alternative as a sensitivity analysis. For Rocha et al. [22], who stimulated on three consecutive days, we used the first acute session.

Handling of crossover trials. The included crossover trials reported post-condition means and standard deviations but not the within-subject correlation, so paired differences could not be reconstructed. Following Cochrane Handbook guidance [16] and Elbourne et al. [23], such trials were analysed as parallel-group comparisons. This is the recommended conservative default. It ignores within-participant pairing and therefore widens the confidence intervals rather than inflating the type I error rate; it makes the analysis less likely, not more likely, to declare an effect. A within-subject correlation of *r* = 0.7 was applied as a sensitivity analysis to gauge the influence of this assumption.

### 2.5. Risk of Bias and Certainty

Risk of bias was assessed with the Cochrane RoB 2 tool, using the parallel-group and crossover versions as appropriate [24], with particular attention to whether the success of sham blinding had been verified. Certainty of evidence for each outcome category was rated with GRADE [25], and imprecision was judged against the criteria of Guyatt et al. [26].

### 2.6. Effect Measures and Synthesis

The effect measure was Hedges’ *g*, anodal versus sham, oriented so that positive values favour anodal tDCS. Estimates were pooled separately for each outcome category. Random-effects meta-analyses used REML with the Hartung–Knapp adjustment [27]; this is the primary interval reported throughout. REML was chosen on the basis of comparative evidence for between-study variance estimators [28].

The Hartung–Knapp interval can be anti-conservative when τ^2^ = 0 and studies are very few. This applies to the three-study kinetics analysis. We therefore also computed a conservative variance-corrected interval, in which the standard error is not allowed to fall below the random-effects standard error [29]. Both intervals are reported.

Heterogeneity was quantified with τ^2^ and I^2^. Prediction intervals are reported where the between-study variance is estimable; for the three-trial kinetics analysis, τ^2^ was zero, so the prediction interval collapses onto the confidence interval and is not reported [30]. Sensitivity analyses used *r* = 0.7, leave-one-out, and—for Chen et al. [21]—the alternative 2 mA condition. The protocol also specified robust variance estimation for outcomes nested within trials. No trial contributed more than one comparison to either outcome category, so no such dependency arose, and the standard random-effects model was used. Publication-bias tests and meta-regression were pre-specified only for ten or more studies and were therefore not performed; below that threshold, such tests cannot be interpreted reliably [31]. For the *r* = 0.7 analysis, the sampling variance of each crossover comparison was multiplied by 2(1 − r). We adopted this convention so that the primary analysis, at *r* = 0.5, reproduces the parallel-group approximation exactly. It is more conservative than the standard change-score variance, 2(1 − r)/n + g^2^/2n, and therefore does not narrow the intervals as much as that formula would.

Primary analyses were performed in R 4.6.0 (R Foundation for Statistical Computing, Vienna, Austria) with the metafor package, version 5.0.1 [32]. To confirm computational reproducibility, both pooled estimates were independently re-derived in Review Manager Web (Cochrane, London, UK; revman.cochrane.org) under the same specification: inverse-variance random effects, REML between-study variance, and the Hartung–Knapp–Sidik–Jonkman interval. The two programs agreed. For jump output, Review Manager returned 0.43 (95% CI −0.05 to 0.92) against 0.44 (−0.05 to 0.92) in metafor; for force–time kinetics, both returned 0.67 (0.53 to 0.81). Review Manager independently flagged the force–time–kinetics analysis as one in which τ^2^ = 0 calls for care in choosing the interval method—the concern our conservative variance correction addresses.

## 3. Results

### 3.1. Study Selection

The search identified 765 records (PubMed 255; Embase 383; Web of Science 76; CENTRAL 51). After 197 duplicates were removed, 568 unique records underwent title and abstract screening. Of these, 523 were excluded (clinical or rehabilitation populations, 211; no explosive lower-limb power outcome, 135; reviews, meta-analyses or protocols, 76; non-anodal or non-M1 stimulation, 41; other, 60).

**Figure 1:**
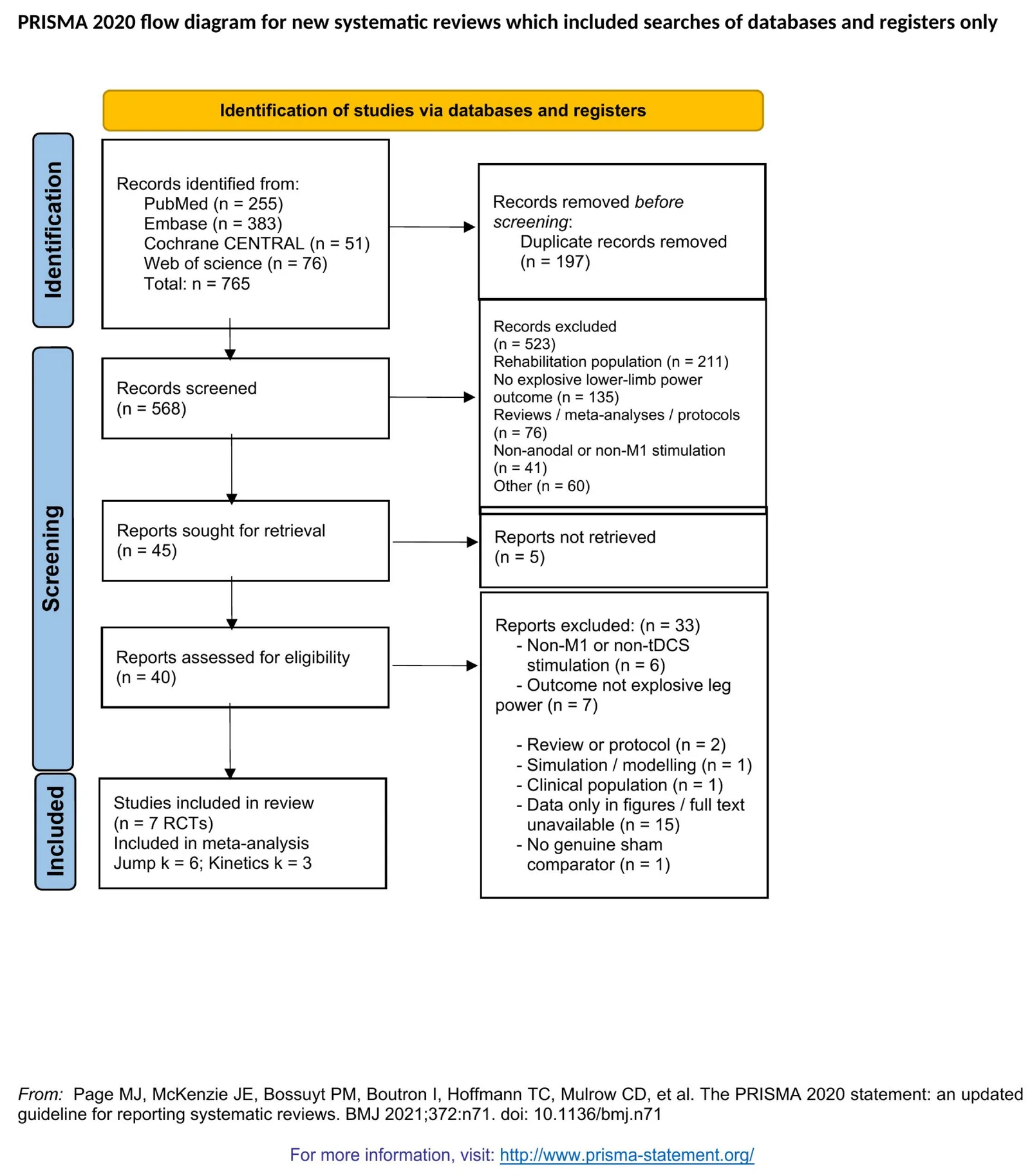
PRISMA 2020 flow diagram of study identification, screening and inclusion. The flow-diagram template is reproduced from Page et al. [14] under the terms of the Creative Commons Attribution 4.0 International licence (CC BY 4.0); the data shown are those of the present review. M1, primary motor cortex; RCT, randomised controlled trial; tDCS, transcranial direct current stimulation.

Forty-five reports were sought for retrieval; five could not be obtained in full text, as they were all behind paywalls without institutional access. Of the 40 reports assessed for eligibility, 33 were excluded. The reasons were: non-M1 or non-tDCS stimulation, 6; outcome not explosive lower-limb power, 7; review or protocol, 2; simulation or modelling, 1; clinical population, 1; outcome data reported only in figures or full text not obtainable, 15; and no genuine sham comparator, 1. Seven trials met all criteria and provided extractable data. Six contributed to jump output and three to force–time kinetics, with two trials contributing to both (Figure 1).

The trial excluded for want of a sham comparator was Zhou et al. [18]. It had four conditions: a no-treatment control, high-load resistance training, tDCS, and tDCS combined with resistance training. The authors state in their own abstract that “no intervention was administered in the control group”, and their Methods describe that condition as an electroencephalography test, a warm-up and a jump test with no device worn. Blinding of the experimenter, not of participants, is what their single-blind design refers to; and they identify the absence of blinding as a limitation of their trial. Because the registered protocol requires a genuine sham condition, the trial was excluded from the primary analyses. Its influence is quantified in Section 3.6.

### 3.2. Study Characteristics and Risk of Bias

Seven trials were included [19,20,21,22,33,34,35]. Five were randomised crossover trials and two were parallel-group trials. Participants were healthy active adults, with 9 to 19 individuals per condition (Table 2). Characteristics of the samples varied widely: professional female volleyball players [35], trained male soccer players [22], female collegiate basketball athletes [21], resistance-trained men [33], recreationally active adults [20,34], and healthy non-athlete men [19]. No trial reported weekly training hours. Table 2 records the training status each report gave, from recreationally active to professional, and we have not inferred a volume that no trial states.

**Figure 2:**
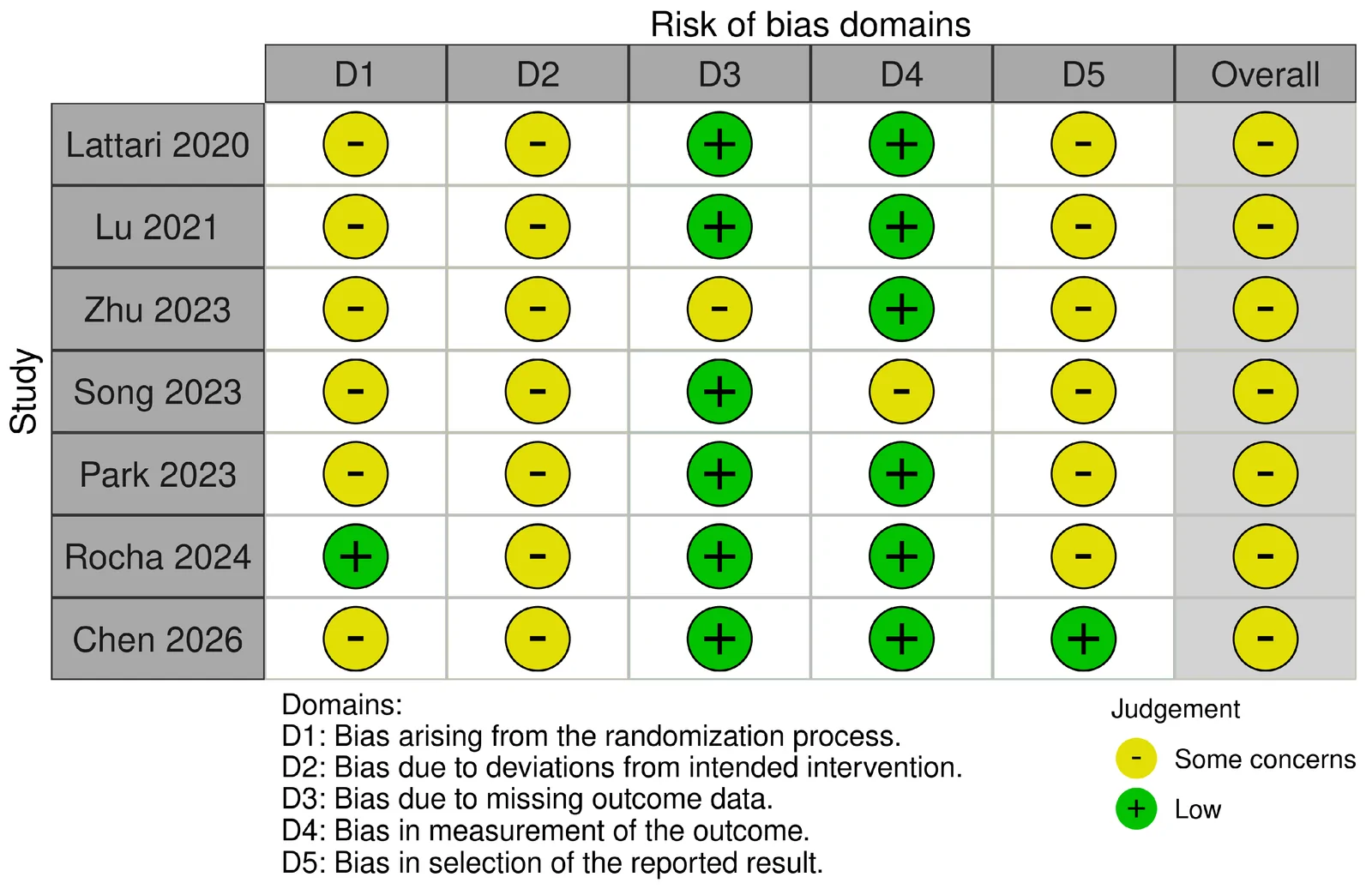
Risk-of-bias (RoB 2) [24] traffic-light plot for the seven included trials [19,20,21,22,33,34,35] across the five domains (D1–D5) and overall. All trials were rated ‘some concerns’; none was rated low or high risk overall.

All seven trials were rated ‘some concerns’ overall on RoB 2; none was rated low risk, and none was rated high risk (Figure 2). The recurring concerns were unverified success of sham blinding (deviations-from-intended-interventions domain), unreported allocation concealment (randomisation domain), and the absence of a prospective registration or protocol (selective-reporting domain). Missing outcome data were low risk in all trials but one [20]. Objective, device-based measurement of jump and force–time outcomes was low risk in all but one trial. For the crossover trials, period and carryover effects were judged low risk: washout periods were reported for every crossover trial and were adequate relative to the short-lived acute effects of tDCS. Per-domain judgements are shown in Figure 2 and, with the supporting rationale for each domain, in Table S3.

#### 3.2.1. Stimulation Parameters

Stimulation parameters are reported in full in Table 3. Only two trials shared an electrode arrangement.

**Table 3:** Stimulation parameters of the seven included trials.

Study	Device	Montage (Anode → Cathode)	Electrode Area	Current	Current Density	Duration; Sessions	Ramp; Sham Procedure
Lattari 2020 [33]	TCT (Brazil)	Cz, over bilateral M1 → Fp2 (right orbitofrontal)	35 cm^2^ saline sponge	2 mA	0.057 mA/cm^2^	20 min; single acute	NR; sham identical, switched off after 30 s
Lu 2021 [19]	Halo Sport	Cz → C5 and C6 (bihemispheric M1)	28 cm^2^ (6.4 × 4.4 cm)	2 mA	0.071 mA/cm^2^	20 min; single acute	Up over 30 s; sham 0 mA
Zhu 2023 [20]	NR	C3 and C4 (two anodes) → both ipsilateral shoulders (extracephalic)	5 × 7 cm = 35 cm^2^ rubber	2 mA	0.057 mA/cm^2^	20 min; single acute	Up and down over 30 s; sham NR
Song and Yim 2023 [34]	Halo Sport 2	M1 (fixed headset; positions NR)	NR	2–3 mA	NR	20 min; 8 sessions (2/week × 4 weeks)	NR; sham recognisable stimulation for 30 s
Park 2023 [35]	Halo Sport	Cz (vertex) → C5 and C6	NR	2 mA	NR	20 min; single acute	NR; sham-controlled
Rocha 2024 [22]	NR	Cz → inion	6 × 8 cm = 48 cm^2^ rubber	2 mA	0.042 mA/cm^2^	15 min; 3 consecutive days	Up and down over first and last 30 s; sham current interrupted after 30 s
Chen 2026 [21]	NeuSen tES	Multifocal: 3 anodes (P3, FC1, C2) → 5 cathodes (F2, FC4, CP3, CP1, CP4)	3.14 cm^2^ per electrode (8 electrodes)	2 mA and 4 mA (4 mA analysed)	NR	20 min; single acute per condition	NR; sham

cm^2^, square centimetre; C, central; CP, centro-parietal; Cz, central midline; F, frontal; FC, fronto-central; Fp, frontopolar; mA, milliampere; M1, primary motor cortex; NR, not reported; P, parietal; TCT, brand name of the stimulator used by Lattari et al. [33]. Electrode positions follow the international 10–20 system, in which the letter denotes the underlying region and the numeral the position, with ‘z’ indicating the midline, even numbers the right hemisphere and odd numbers the left. Current density was not reported by any trial and was calculated by the present authors where electrode area was given. Transcranial direct current stimulation delivers a constant, non-pulsed current, so waveform, pulse amplitude, pulse width and pulse frequency are not defined for this modality.

Intensity and duration. Five trials delivered 2 mA [19,20,22,33,35]; Song and Yim [34] used 2–3 mA, and Chen et al. [21] compared 2 mA with 4 mA. Six trials stimulated for 20 min; Rocha et al. [22] stimulated for 15 min.

Montage. Six distinct arrangements were used:

• Anode at Cz with the cathode over the right orbitofrontal region (Fp2) [33];
• Anode at Cz with cathodes at C5 and C6 [19,35];
• Two anodes at C3 and C4 with extracephalic cathodes over both shoulders [20];
• Anode at Cz with the cathode at the inion [22];
• A multifocal eight-electrode arrangement optimised by finite-element modelling, with three anodes and five cathodes [21];
• A fixed-electrode consumer headset, for which electrode positions were not specified [34].

Electrode area and current density. Electrode area ranged from 3.14 cm^2^ per electrode in the multifocal montage [21]  to 48 cm^2^   [22]. Where area was reported, current density ranged from 0.042 to 0.071 mA/cm^2^. No trial reported current density directly; we calculated it. Two trials did not report electrode dimensions [34,35].

Ramping and sham procedure. Three trials described ramping the current over 30 s [19,20,22];  two of them specified ramping at both onset and offset [20,22].   Sham was implemented by applying the electrodes and discontinuing the current after approximately 30 s [22,33,34],  the standard approach [36].

Dose and sessions. Five trials delivered a single acute session before testing. Rocha et al. [22]  stimulated on three consecutive days; Song and Yim   [34]  stimulated twice weekly for four weeks alongside a plyometric programme. Three trials used commercial fixed-montage headsets of the Halo Sport type   [19,34,35],  whose electrode configuration cannot be individualised.

We note that transcranial direct current stimulation delivers a constant, non-pulsed current—described in one included trial as a “monophasic continuous current” [20]. Waveform, pulse amplitude, pulse width, and pulse frequency are therefore not defined for this modality; they belong to pulsed or alternating techniques. The parameters that determine tDCS dose are current intensity, current density, electrode size and position, ramping, stimulation duration, and the timing of stimulation relative to the task [36,37]. All are reported in Table 3.

#### 3.2.2. Motor Performance Assessments

Assessment procedures are reported in Table 4. Measurement methods were not uniform.

**Table 4:** Motor performance assessments in the seven included trials.

Study	Outcome Entered into Meta-Analysis	Test	Measurement Instrument	Trials; Score Used	Assessment Time Points
Lattari 2020 [33]	CMJ height (jump)	CMJ, hands on hips	Contact mat; kinematics filmed at 120 Hz	3 trials; best (ICC 0.97)	Pre- and post-stimulation
Lu 2021 [19]	Rate of force development, knee extensor (kinetics)	Isometric maximal voluntary contraction, instructed to reach maximal force as fast as possible	Isokinetic dynamometer with surface electromyography	NR	Before, immediately after, and 30 min after
Zhu 2023 [20]	CMJ height (jump); knee peak power (kinetics)	CMJ	Two Kistler 9287B force plates at 1200 Hz with Vicon T40 motion capture (240 Hz)	NR	Pre, immediately post, and 30 min post
Song and Yim 2023 [34]	Vertical jump height (jump)	Jump-and-reach against a wall; fingertip mark, difference recorded in cm	Wall mark and tape measure	NR	Before and after the 4-week programme
Park 2023 [35]	CMJ height (jump)	CMJ and jump-and-reach	NR	3 trials; highest	Pre and post
Rocha 2024 [22]	CMJ height (jump)	CMJ	NR	3 repetitions	After warm-up and 15 min post-stimulation, on each of 3 days
Chen 2026 [21]	CMJ height (jump); relative peak power (kinetics)	CMJ, SJ and DJ; jump height derived from take-off velocity	Kistler force platform with MARS software and surface electromyography	NR	Baseline and after each stimulation session

CMJ, countermovement jump; DJ, drop jump; ICC, intraclass correlation coefficient; NR, not reported; SJ, squat jump. Measurement method varied: two trials used force platforms, one a contact mat, one an isokinetic dynamometer, and one a wall-mark jump-and-reach without instrumented force measurement.

Two trials measured jumps on three-dimensional force platforms. Zhu et al. [20] used two Kistler 9287B plates sampling at 1200 Hz with synchronised motion capture. Chen et al. [21] used a Kistler platform, deriving jump height from take-off velocity. Lattari et al. [33] used a contact mat with video at 120 Hz. Lu et al. [19] measured isometric maximal voluntary contraction and RFD of the knee extensors and flexors on a dynamometer with surface electromyography. Song and Yim [34] measured jump height by marking the fingertips against a wall, a jump-and-reach method without instrumented force measurement. Park et al. [35] and Rocha et al. [22] did not report the measurement instrument.

Three trials reported the number of attempts and the score taken: the best of three trials [33,35] and three repetitions [22]. Four did not. Assessment timing also differed. Five trials tested immediately after or within 15 min of stimulation; Zhu et al. [20] and Lu et al. [19] additionally tested 30 min afterwards, and Song and Yim [34] tested before and after a four-week programme.

This variation matters for interpretation. The trial with the least precise measurement method [34] produced one of the largest individual effects (*g* = 0.90), but so did one of the two force-platform trials (*g* = 0.97); the other gave 0.47. The measurement method is therefore a candidate source of the heterogeneity observed in the jump analysis.

### 3.3. Jump Output

**Figure 3:**
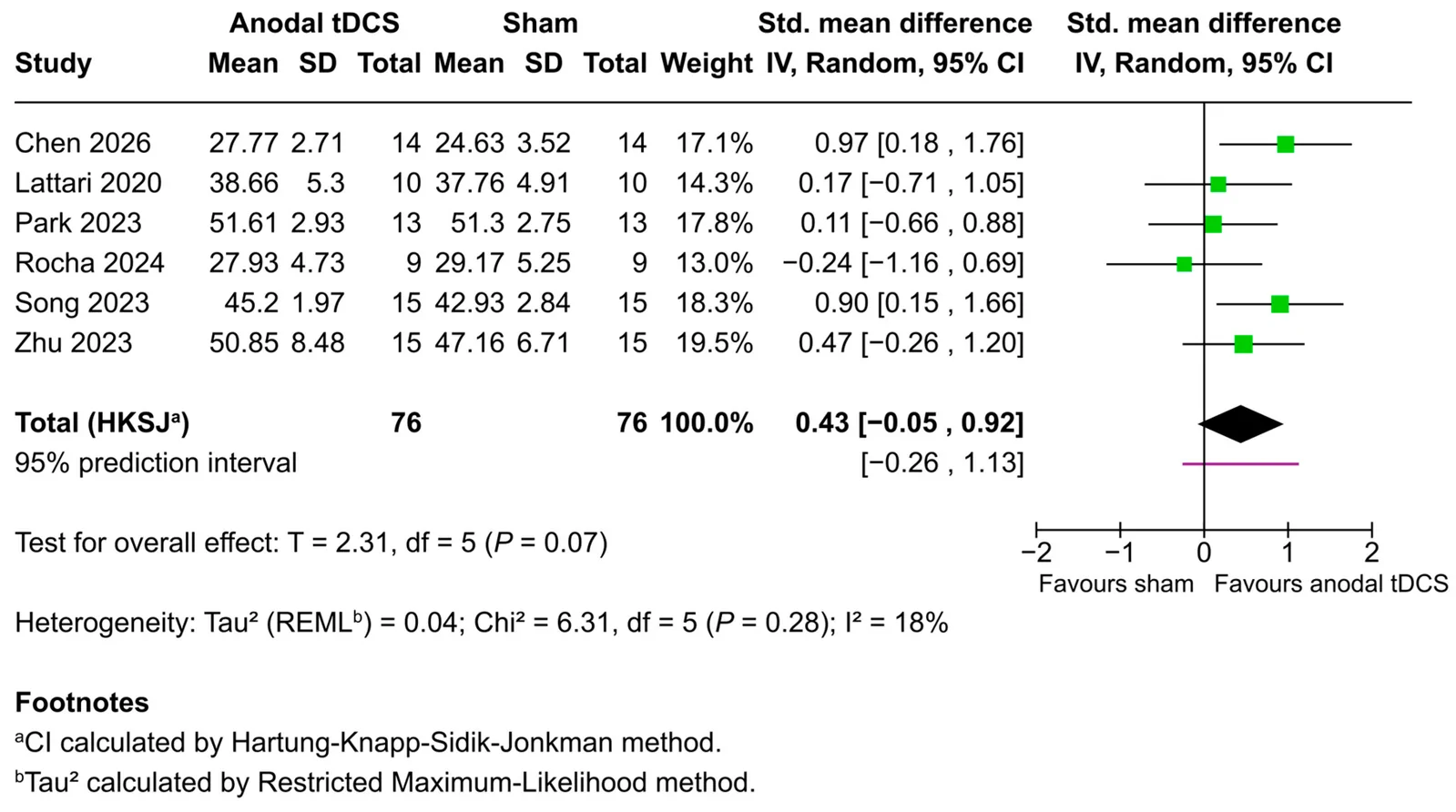
Forest plot of anodal M1 tDCS versus sham on jump output (*k* = 6) [20,21,22,33,34,35]. Squares are study-level Hedges’ *g*, sized by inverse-variance weight; horizontal lines are 95% confidence intervals; the diamond is the random-effects pooled estimate (REML with the Hartung–Knapp–Sidik–Jonkman interval [27]): *g* = 0.44, 95% CI −0.05 to 0.92. The 95% prediction interval [30] was −0.27 to 1.14. The plot is Review Manager’s output, whereas the text reports metafor’s [32]. Three values therefore differ in the last digit shown: the pooled estimate (0.43 against 0.44), the prediction interval (−0.26 to 1.13 against −0.27 to 1.14), and I^2^ (18% against 19.1%). We have not been able to reconcile the two I^2^ values against either program’s documented formula, and we report the difference rather than explain it. The two derivations are available from the corresponding author on request.

Pooling six comparisons, anodal M1 tDCS did not significantly improve jump output relative to sham (Hedges’ *g* = 0.44, 95% CI −0.05 to 0.92; *p* = 0.069; *I*^2^ = 19.1%, τ^2^ = 0.039). The 95% prediction interval was −0.27 to 1.14. Individual estimates ranged from −0.24 to 0.97 (Figure 3).

Leave-one-out analysis gave pooled estimates between 0.33 and 0.54; the result reached significance only when Rocha et al. [22], the single trial with a negative point estimate, was omitted (*g* = 0.54, 95% CI 0.05 to 1.03; *p* = 0.038). The estimate was essentially unchanged under a within-subject correlation of *r* = 0.7 (*g* = 0.43, 95% CI −0.04 to 0.90; *p* = 0.064). Substituting the 2 mA condition of Chen et al. [21] for the 4 mA condition reduced the pooled estimate and removed all measurable heterogeneity (*g* = 0.33, 95% CI −0.06 to 0.72; *p* = 0.081; *I*^2^ = 0%). The two parallel-group trials produced the lowest (*g* = −0.24) and the second-highest (*g* = 0.90) of the six estimates, so the estimates do not separate by design. With only two parallel-group trials, a formal comparison would not be interpretable, and none is reported.

Two of the six comparisons came from parallel-group trials, in which randomisation can leave the arms unbalanced before treatment. After the leave-one-out analysis identified Rocha et al. [22] as influential, we returned to that report and examined its baseline values. The arms differed by 1.29 cm before stimulation (28.93 cm anodal, 30.22 cm sham), and the post-intervention difference of 1.24 cm is almost exactly that pre-existing gap: the two arms changed by −1.00 cm and −1.05 cm, respectively. The negative estimate contributed by this trial therefore reflects baseline imbalance rather than a negative response to stimulation. We therefore repeated the analysis with baseline-adjusted change scores for the two parallel-group trials; the crossover trials need no such adjustment, since each participant serves as their own control. That gave *g* = 0.49 (95% CI 0.03 to 0.95; *p* = 0.041). Adjusting Rocha et al. alone gave *g* = 0.47 (95% CI 0.04 to 0.90; *p* = 0.037). This analysis was not pre-specified and was prompted by the leave-one-out result, so we report it as a post hoc sensitivity analysis and not as a primary finding; the registered protocol specified post-intervention means and standard deviations. It is nonetheless a real fragility, and it runs in the opposite direction to the exclusion reported in Section 3.6.

### 3.4. Force–Time Kinetics

**Figure 4:**
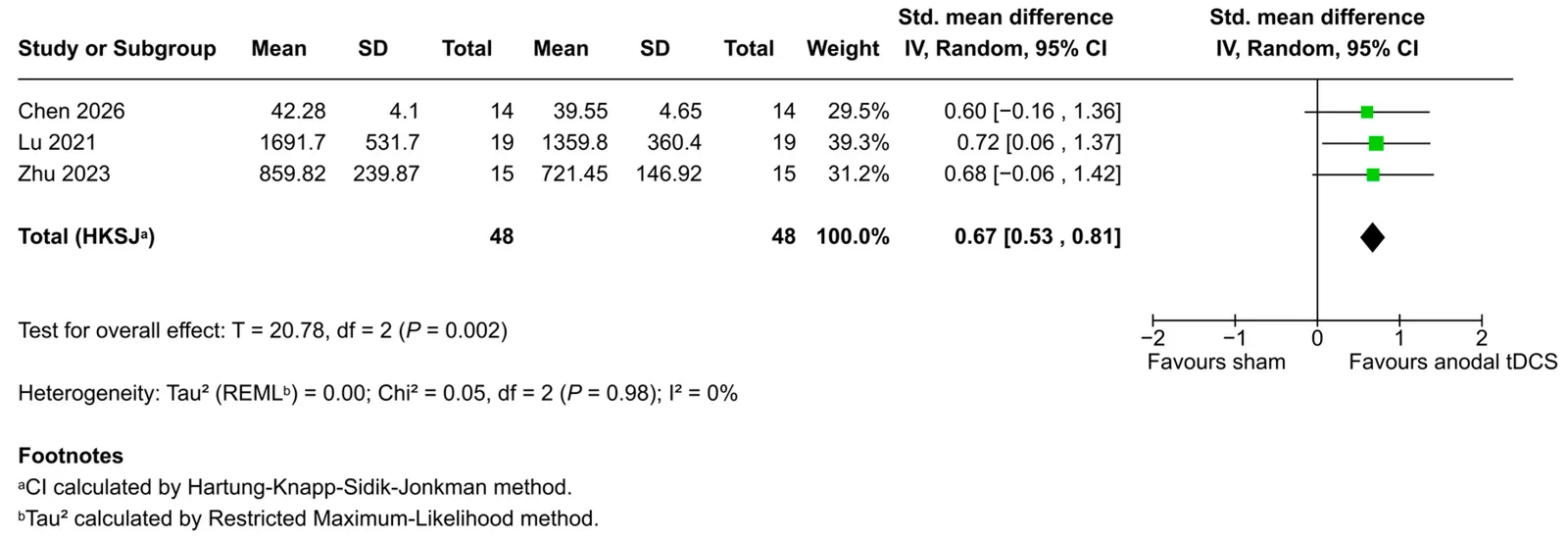
Forest plot of anodal M1 tDCS versus sham on force–time kinetics (*k* = 3) [19,20,21]. The diamond is the random-effects pooled estimate (REML with the Hartung–Knapp–Sidik–Jonkman interval [27]): *g* = 0.67, 95% CI 0.53 to 0.81. Because that interval can be anti-conservative with only three trials and zero heterogeneity, a conservative small-sample variance correction [29] was also applied: 95% CI −0.23 to 1.57, *p* = 0.086. The effect is not statistically significant under the corrected interval. As in Figure 3, the study-level values printed here are Review Manager’s: Chen et al. [21] appears as 0.60 against 0.61 in the text, the same rounding difference.

Pooling three comparisons, the estimate for force–time kinetics was moderate (Hedges’ *g* = 0.67, 95% CI 0.53 to 0.81; *p* = 0.002; *I*^2^ = 0%; REML with Hartung–Knapp) (Figure 4). Individual effects were closely clustered (0.61, 0.68, 0.72).

This interval must be read with caution, and one feature makes the reason plain. The pooled Hartung–Knapp standard error is 0.032, whereas the standard errors of the three contributing trials are 0.335, 0.375 and 0.387. An interval an order of magnitude narrower than any of its constituents is not credible on its face; it is the signature of the Hartung–Knapp adjustment becoming anti-conservative when τ^2^ = 0 and trials are few. Under the conservative variance correction, the effect was no longer significant (95% CI −0.23 to 1.57; *p* = 0.086) [29]. The same pattern held under *r* = 0.7 (corrected 95% CI −0.03 to 1.37; *p* = 0.054). Leave-one-out estimates ranged from 0.64 to 0.70. With only three trials, the observed *I*^2^ = 0% more plausibly reflects low power to detect inconsistency than genuine homogeneity. We regard the corrected interval as the one on which any inference should rest.

The joint from which that estimate is taken matters as much as the choice of trial. Zhu et al. [20] report peak power for the ankle, knee and hip; we used the knee. Substituting hip power moves the pooled estimate to *g* = 0.50 (95% CI −0.03 to 1.02; *p* = 0.11), and ankle power moves it to *g* = 0.43 (95% CI −0.27 to 1.13; *p* = 0.22; *I*^2^ = 25%). Neither substitute is more defensible than the value we used because the registered protocol names peak or mean power without naming a joint. The point is that this estimate, and the zero heterogeneity that accompanies it, rest on a selection that the protocol did not make.

### 3.5. Comparison Between Outcome Categories and Certainty

Force–time kinetics gave the larger point estimate (*g* = 0.67 versus 0.44), the direction anticipated in our hypothesis. Their confidence intervals overlapped substantially, no formal between-category test was performed, and two trials [20,21] contributed to both estimates, so the categories are not statistically independent. Neither estimate was robust: jump output was not significant, and the kinetics estimate did not survive the conservative correction. No dissociation can be claimed.

Fewer than ten trials contributed to each analysis, so publication-bias tests were not performed; below that threshold, funnel-plot and regression-based tests cannot be interpreted reliably [31]. Small-study effects nonetheless remain a serious concern. GRADE certainty was very low for both outcome categories, downgraded for risk of bias, indirectness, and imprecision (Table 5; full evidence profile in Table S4). Imprecision was rated serious for both: the confidence interval for jump output includes both no effect and a large benefit, and the kinetics interval does so once the small-sample correction is applied [26].

### 3.6. Post Hoc Sensitivity Analysis: Inclusion of the Non-Sham Trial

Zhou et al. [18] was excluded because its comparator was a no-intervention control. Because that trial was the largest in the evidence base (29 participants per condition) and contributed to both outcome categories, we quantified its influence post hoc.

Adding it rendered both estimates significant: jump output *g* = 0.55 (95% CI 0.11 to 0.98; *p* = 0.022; *k* = 7) and force–time kinetics *g* = 0.77 (conservative 95% CI 0.24 to 1.30; *p* = 0.020; *k* = 4). In other words, the statistical significance of this evidence base depends on a single trial that lacked a sham comparator and whose authors reported no blinding. We regard this as a further indication of how fragile the current evidence is, and we report it so that readers may judge it for themselves.

## 4. Discussion

### 4.1. Principal Findings

This review asked a narrow question. In healthy active adults, does anodal M1 tDCS improve explosive lower-limb power relative to sham? We analysed jump output and force–time kinetics separately because the two capture different levels of the same capacity.

The answer, on present evidence, is that it does not do so reliably. Jump output showed a small-to-moderate average estimate favouring stimulation, but the interval crossed zero. The prediction interval was wider still, so a future trial drawn from this literature could plausibly return a null or negative result. Force–time kinetics gave a larger and more consistent estimate. That consistency did not survive scrutiny: with only three contributing trials, the interval widened to include zero once a conservative small-sample correction was applied.

Their direction matched our hypothesis, in that the kinetic estimate exceeded the jump estimate. We place little weight on this. Intervals overlap, two trials contribute to both analyses, and neither estimate is statistically robust. We treat the ordering as a hypothesis for future work, not a finding.

To our knowledge, this is the first synthesis to separate jump displacement from force–time kinetics under sham-controlled conditions. We regard the separation itself as the more durable contribution. It reduces outcome-level heterogeneity, and it makes visible a distinction that whole-domain pooling obscures.

One further point bears on how much confidence any of this deserves. A trial we excluded for want of a sham comparator would, had it been retained, have rendered both estimates significant. An evidence base whose conclusions turn on the inclusion of a single unblinded trial is not one from which firm inferences can be drawn.

### 4.2. Comparison with Existing Literature

Our estimates sit within the range reported by earlier syntheses, though at their lower end once instability is taken into account. Winker et al. found a small average effect of M1 tDCS on athletic performance, with high heterogeneity, and identified jumping among the more responsive tasks [9]. Machado et al. and Holgado et al. converged on small pooled effects with substantial inconsistency, the former finding no reliable strength benefit [7,8]. Alix-Fages et al. reached a similarly guarded conclusion for maximal force and endurance [13]. Chinzara et al. reported benefits that varied by domain [11]. Maudrich et al., restricting to single-session protocols in athletes, found a moderate effect that was clearest in visuomotor tasks [10].

Most instructive is the umbrella review by Holgado et al. [12]. Synthesising nine meta-analyses covering fifty crossover studies, the authors found that the average effect did not survive correction for publication bias. Our null result for jump output is consistent with that conclusion rather than at odds with it.

The populations and sports differ substantially across this literature and across our own included trials. Ours span professional female volleyball players [35], trained male soccer players [22], female collegiate basketball athletes [21], men with several years of resistance-training experience [33], recreationally active adults [20,34], and healthy non-athlete men [19]. Prior syntheses pooled wider samples still, including endurance athletes and untrained volunteers. This matters in two ways. Trained athletes may have less headroom for acute neural facilitation than untrained participants, which would attenuate effects in the very samples where an ergogenic aid would be marketed. And the sports represented here are jump-dominated, whereas earlier reviews drew heavily on cycling and running protocols in which the relevant motor demand is quite different. Our narrower scope therefore isolates a more homogeneous phenotype, but at the cost of a small evidence base.

Measurement differs too. Only two of our trials used force platforms [20,21]; one measured jump height by a fingertip mark against a wall [34]. Earlier reviews rarely stratified by measurement precision. When effect sizes of this magnitude are at stake, the difference between an instrumented platform and a wall mark is not trivial.

Where we differ from earlier work is chiefly in precision, and that difference is a weakness rather than a strength. Our estimates rest on very few trials per outcome category and were graded as very low certainty. The larger prior reviews drew on more trials and produced more precise, if more heterogeneous, estimates. Their more conservative conclusions should be given corresponding weight.

### 4.3. Mechanistic Interpretation

If anodal M1 tDCS confers any ergogenic benefit, the most plausible pathway is a transient rise in corticospinal excitability and descending neural drive. Anodal stimulation depolarises the resting membrane potential of cortical neurons. In the after-effect window, it enhances corticospinal output through mechanisms with a partly N-methyl-D-aspartate (NMDA)-receptor-dependent, plasticity-like signature [6,37].

A larger or more synchronous volley to the spinal motoneuron pool would raise motor-unit recruitment and discharge rate at the onset of contraction. That is the phase governing early RFD. The first tens of milliseconds of an explosive contraction are dominated by neural rather than contractile determinants. Peak force and peak power, by contrast, reflect a broader mix of muscle–tendon and morphological factors. Any stimulation-induced gain in neural drive should therefore appear most clearly in early-phase kinetic variables.

Our data are compatible with that ordering, but only weakly. Force–time kinetics gave the numerically larger estimate and showed no detectable heterogeneity. Neither observation is secure. Intervals for the two outcome categories overlap under either interval method. No formal between-category contrast was performed. Two trials contribute to both estimates, so the categories are not independent. Measurement level, study overlap, or chance could equally explain the apparent separation.

Jump height may also absorb an upstream neural gain. It sits at the distal end of a long kinetic chain. Hip, knee and ankle torques, stretch-shortening-cycle efficiency, tendon energy storage, inter-joint coordination and technique all intervene between cortical drive and whole-body output [2]. Each can buffer a change before it reaches the measured endpoint. Ground-reaction-force kinetics are recorded closer to the point of force application and may retain a cleaner signature.

What dose would be required? The honest answer is that the present evidence cannot specify one. Four of the seven trials converged on 2 mA for 20 min in a single session—a dose whose cortical after-effects are typically short-lived and modest [36,37]. Such protocols are unlikely to engage the cumulative, plasticity-driven adaptations that longer paradigms might recruit. The only within-trial dose comparison available is that of Chen et al. [21], who found 4 mA effective where 2 mA was not; when we substituted their 2 mA condition into our analysis, the pooled estimate fell. That single comparison cannot establish a dose–response relationship. Current density, which determines the field actually delivered, was reported by no trial and varied by nearly two-fold once we calculated it. Until trials report density and vary intensity deliberately, no defensible dosing recommendation can be made.

Montage varied widely across trials, encompassing conventional bilateral arrangements, extracephalic returns, multifocal configurations, and fixed-electrode consumer headsets. The cortical territory that was actually modulated therefore differs between trials, and its relevance to lower-limb motor representations is often uncertain. Given very-low-certainty evidence, few trials, single-assessor screening, and the analysis of crossover trials as parallel-group comparisons, these mechanistic accounts should be read as generating testable hypotheses. Chief among them is that early-phase RFD is the outcome most sensitive to anodal M1 tDCS. None of them establishes a causal or dose-dependent mechanism. Wider work supports this caution, reporting small, montage- and task-moderated effects that attenuate once power and reporting biases are considered [7,8,9,12,13].

### 4.4. Moderators and Sources of Heterogeneity

Six trials contributed to the jump analysis and three to the kinetics analysis. Both fall well below the ten-studies-per-covariate threshold conventionally required before subgroup analysis or meta-regression can be interpreted. We therefore tested no moderators formally. What follows is description, not evidence of effect modification.

Training status shows no consistent gradient in these data. Sizeable positive estimates arose in trained female athletes [21] and in recreationally active samples [34]; the only negative estimate arose in trained male soccer players [22]. Training status is confounded with intervention duration, montage, and sex, so nothing can be disentangled. In particular, these data do not support a simple ceiling pattern in which more highly trained systems respond less.

Dose and design are likewise inseparable here. The two multi-session trials [22,34] differed from one another and from the acute crossover trials in participants and co-interventions. Song and Yim [34] embedded stimulation within a four-week plyometric programme, so any benefit cannot be attributed to stimulation alone.

Sex could not be examined. Two trials enrolled women exclusively [21,35]; the remainder were male-only or male-predominant.

Measurement method deserves particular attention, and Table 4 makes it visible. The trial using a wall-mark jump-and-reach [34] produced the second-largest estimate (*g* = 0.90), but the largest came from a force-platform trial [21] (*g* = 0.97), so measurement method does not order the estimates on its own. We cannot test this formally. It is, however, a more concrete candidate explanation for the observed inconsistency than the moderators usually invoked.

### 4.5. Limitations

Our evidence base is small: six trials for jump output and three for force–time kinetics. Formal publication-bias assessment and meta-regression were therefore impossible, and small-study effects remain strongly suspected [12,31]. Samples were small and designs were predominantly acute and single-session. Sham blinding was rarely verified, allocation concealment seldom reported, and prospective registration infrequent; all seven trials carried some concerns on RoB 2.

Four deviations from the registered protocol should be noted. First, two of six pre-specified databases could not be accessed, mitigated by citation tracking. Second, screening and extraction were conducted primarily by one assessor rather than two, and Cohen’s kappa is therefore not reported; the verification audit described in Section 2.4 was added to quantify the consequences. Third, the crossover trials were analysed as parallel-group comparisons because within-subject correlations were unavailable—a conservative approximation that widens their intervals [16,23]. Fourth, the protocol specified a narrative summary for any outcome category containing fewer than four trials. The force–time–kinetics category meets that threshold only with the non-sham trial included (*k* = 4, reported post hoc in Section 3.6); without it, *k* = 3. We report the pooled estimate for continuity with that analysis but treat it as descriptive rather than confirmatory: it does not survive the conservative variance correction, and its certainty is very low. For the same reason, we report no subgroup comparison by trial design, which would have rested on two parallel-group trials.

For one trial that was excluded after re-examination of every comparator condition, we established that it had used a no-intervention control rather than sham [18]. The registered protocol treats the absence of a genuine sham as an exclusion criterion, so this correction restores rather than departs from the protocol. We report its effect on the pooled estimates in Section 3.6 so that the consequence is fully visible.

Retrieval failure is a further limitation, and one we can make concrete rather than leave abstract. Five reports could not be obtained in full text, and a larger group reported outcomes only in figures. The verification audit identified several of these as probably eligible, and we name them here because their direction matters.

Mesquita et al. compared bi-hemispheric anodal M1 tDCS with sham in nineteen taekwondo athletes and reported no difference in countermovement jump performance [38]. Jung et al. compared active with sham tDCS alongside six weeks of physical training in fifty-six healthy adults and found no between-group difference in Sargent jump [39]. Codella et al. examined bihemispheric anodal M1 tDCS against sham in seventeen physically active men and reported a 5% increase in lower-limb power [40].

Two of these three unretrieved trials reported null findings. Had they been available, they would most plausibly have pulled the pooled estimate downwards. Our already non-significant result may therefore still be optimistic. We were able to retrieve a fourth trial, Liu et al. [41], but it reports its jump outcomes only in figures and so could not be entered either. With six trials in the jump analysis, one or two additions could shift it appreciably. Overall GRADE certainty was very low for both outcome categories.

### 4.6. Future Directions

The most pressing need is for adequately powered, prospectively registered trials. Typical enrolments here were around a dozen participants, which leaves individual estimates fragile and the pooled effect unstable.

The required scale is easily stated. Detecting a between-condition difference of g ≈ 0.8 in a parallel design, with 80% power at a two-sided alpha of 0.05, needs roughly 25 participants per arm. At g ≈ 0.5, it needs about 63 per arm. Anchored to the smaller effect seen in broader syntheses (g ≈ 0.29), it would need on the order of 190 per arm [9]. An efficient crossover requires fewer, but still far more than are currently available.

Reaching ten trials per outcome category would also permit meta-regression for the first time, and with it a test of whether montage, current density, or training status moderate the response. Protocols should be registered a priori with pre-specified primary outcomes, should report exact within-subject correlations for repeated-measures data, and should be powered to the plausible effect rather than to the largest reported one.

Methodological rigour around blinding would strengthen confidence further. Blinding of participants and personnel drove the risk-of-bias concerns throughout this review. Future trials should use validated sham protocols with explicit integrity checks—post-session allocation guesses and adverse-sensation questionnaires—and report those outcomes. Active and sham tDCS can produce distinguishable cutaneous sensations, and expectancy may plausibly contribute to any performance benefit.

Standardised reporting of kinetic indices alongside jump height would also help, using harmonised epochs and normalisation. So would deliberate variation of dose and montage: head-to-head comparisons of conventional, extracephalic, bi-hemispheric and multifocal arrangements, explicit dose–response arms, and multi-session paradigms combined with structured training. Current density should be reported as a matter of course. Until such trials accumulate, the present findings remain hypothesis-generating.

### 4.7. Practical Applications

The practical message is straightforward, and it has changed from what an earlier reading of this literature might have suggested. Anodal M1 tDCS cannot be regarded as an evidence-based ergogenic aid for explosive lower-limb power. Our primary analysis found no significant effect on jump output, and the kinetic estimate did not withstand a conservative correction. Coaches and practitioners should treat any current use as experimental.

Several considerations reinforce that position. Acute single-session protocols are by far the most studied configuration; durability, interaction with training, and behaviour across repeated exposures remain poorly characterised. What few multi-session designs exist were confounded by co-administered training, so a specific contribution of stimulation cannot be isolated. Much of the applied literature relies on commercial fixed-montage headsets whose electrode configuration is not individualised and whose cost may limit equitable access.

Blinding and expectancy deserve emphasis here. Transient scalp sensations can compromise masking, and sham conditions do not always conceal allocation. Expectancy effects are plausible contributors to any observed improvement; tDCS has been shown to affect subjective as well as objective performance indices [8]. Every trial in this review carried some concerns on the blinding domain, and none verified blinding integrity.

tDCS should therefore not be positioned as a substitute for established methods of developing explosive power—structured strength, plyometric and velocity-based training. At most it might be explored as an experimental adjunct, in monitored and ethically supervised settings, on the explicit understanding that current evidence neither confirms a reliable benefit nor establishes a mechanism.

## 5. Conclusions

We hypothesised that anodal M1 tDCS would improve explosive lower-limb power relative to sham, and that any effect would be larger for force–time kinetics than for jump output.

The first hypothesis was not supported. Jump output showed no significant benefit, and the moderate estimate for force–time kinetics did not survive a conservative small-sample correction. The second received only weak, non-independent support: the kinetic estimate exceeded the jump estimate, but their intervals overlapped, and two trials contributed to both.

Certainty was very low throughout, and the significance of this evidence base proved to depend on a single trial that lacked a sham comparator. Separating jump output from force–time kinetics remains, in our view, the right analytical approach. Applying it to an adequately powered, properly blinded evidence base is the work that remains.

## Figures and Tables

**Table 1:** Eligibility criteria.

Domain	Included	Excluded
Population	Healthy adults aged 18–45 years, from recreationally active to trained. We applied ‘healthy’ operationally, as the included trials themselves did: participants described by the authors as free of injury or illness at the time of testing and not recruited on the basis of a clinical condition. No trial reported a formal health screen, and we did not impose one beyond the exclusions opposite.	Clinical or rehabilitation populations; older adults; sedentary-only samples; children and adolescents
Intervention	Anodal tDCS over the primary motor cortex (M1), any montage; acute or multi-session	Non-M1 targets; cathodal-only stimulation; tACS; tsDCS; temporal interference stimulation; transcranial magnetic stimulation
Comparator	Sham (placebo) tDCS, with electrodes applied and the current ramped and then discontinued	Studies without a genuine sham condition, including no-intervention or resting controls
Outcomes	Two pre-specified categories, analysed separately. Jump output: CMJ, CMJA, SJ or DJ height or distance. Force–time kinetics: rate of force development, peak or mean power, peak force	Endurance, upper-limb, cognitive or sport-technical outcomes only
Study design	Randomised controlled trials, parallel-group or randomised crossover	Non-randomised designs; single-arm studies; reviews and protocols; simulation studies; abstracts without extractable data
Reporting	Post-intervention mean and standard deviation, or a convertible statistic, for both conditions	Data presented only in figures without recoverable values; full text not retrievable
Language, date	No restriction	—

—, no exclusion applied; CMJ, countermovement jump; CMJA, countermovement jump with arm swing; DJ, drop jump; M1, primary motor cortex; SJ, squat jump; tACS, transcranial alternating current stimulation; tDCS, transcranial direct current stimulation; tsDCS, transcutaneous spinal direct current stimulation. Criteria are those of the registered protocol (PROSPERO CRD420261438841).

**Table 2:** Characteristics of the seven included trials.

Study	Country	Design	*n* per Condition	Population	Comparator	Outcome Category	RoB 2 Overall
Lattari 2020 [33]	Brazil	Crossover	10	Men with advanced strength-training experience	Sham (device switched off after 30 s)	Jump	Some concerns
Lu 2021 [19]	China	Crossover	19	Healthy young non-athlete men	Sham (0 mA)	Kinetics	Some concerns
Zhu 2023 [20]	China	Crossover	15	Recreationally active adults	Sham	Jump, Kinetics	Some concerns
Song and Yim 2023 [34]	Republic of Korea	Parallel	15	Recreationally active adults	Sham (recognisable stimulation for 30 s), with plyometric training in both arms	Jump	Some concerns
Park 2023 [35]	Republic of Korea	Crossover	13	Professional female volleyball players	Sham	Jump	Some concerns
Rocha 2024 [22]	Brazil	Parallel	9	Male trained soccer players	Sham (current interrupted after 30 s)	Jump	Some concerns
Chen 2026 [21]	China	Crossover	14	Trained female collegiate basketball athletes	Sham	Jump, Kinetics	Some concerns

mA, milliampere; RoB 2, Cochrane Risk of Bias 2 tool. Jump, jump output category; Kinetics, force–time kinetics category. Two trials [20,21] contributed to both outcome categories. No trial was rated low or high risk overall. Stimulation parameters are reported in Table 3 and assessment procedures in Table 4.

**Table 5:** GRADE summary of findings.

Outcome Category	Trials (Participants per Condition)	Hedges’ *g* (95% CI)	Certainty	Reasons for Downgrading
Jump output	6 (76)	0.44 (−0.05 to 0.92)	Very low (⊕○○○)	Risk of bias (−1): all trials rated some concerns; success of sham blinding never verified and allocation concealment rarely reported. Indirectness (−1): montage, dose and measurement method differed across trials; three used fixed-montage consumer headsets. Imprecision (−1): *k* = 6, and the interval includes both no effect and a large benefit; the 95% prediction interval was −0.27 to 1.14.
Force–time kinetics	3 (48)	0.67 (0.53 to 0.81) with the Hartung–Knapp adjustment; −0.23 to 1.57 under a conservative small-sample variance correction	Very low (⊕○○○)	Risk of bias (−1): as above. Indirectness (−1): three different measurement systems; outcomes not harmonised. Imprecision (−1): *k* = 3, and the effect does not survive the conservative variance correction, under which the interval includes no effect.

CI, confidence interval; GRADE, Grading of Recommendations Assessment, Development and Evaluation. Both categories began as high certainty (randomised trials) and were downgraded three levels. Publication bias could not be formally assessed because fewer than ten trials contributed to each category; small-study effects are nonetheless suspected. ⊕○○○, very low certainty.

## Data Availability

All data analysed in this review are derived from previously published studies, which are cited in the References. The full search strategies (Table S1), the PRISMA 2020 checklist (Table S2), the Risk-of-Bias (RoB 2) Assessments for the Seven Included Studies (Table S3), and the GRADE Summary of Findings (Table S4) are provided as Supplementary Materials. The extracted study-level dataset, the R (metafor) analysis code, the Review Manager cross-validation and the verification-audit sampling frame are available from the corresponding author on request; every estimate reported here can be reproduced from them. No new primary data were generated.

## Supplementary Materials

The following supporting information can be downloaded at: https://www.mdpi.com/article/10.3390/sports14090401/s1, Table S1: Full electronic search strategies; Table S2: PRISMA 2020 Checklist; Table S3: Risk-of-Bias (RoB 2) Assessments for the Seven Included Studies [19,20,21,22,33,34,35]; Table S4: GRADE Summary of Findings. The extracted study-level dataset, the R (metafor) analysis code, the Review Manager cross-validation and the verification-audit sampling frame are available from the corresponding author on request.
