# Supplementary material for: Anodal Motor-Cortex Transcranial Direct Current Stimulation and Explosive Lower-Limb Power in Healthy Active Adults: A Sham-Controlled Systematic Review and Meta-Analysis
Source: Sports (Basel). 2026 Sep 11;14(9):401. doi: 10.3390/sports14090401 (PMC13611768; doi:10.3390/sports14090401)
Supplement: Supplementary file 1 [file sports-14-00401-s001.zip › Table_S1_Search_strategies.pdf]

Table S1. Full electronic search strategies

Complete keywords, Boolean operators and their combinations for every database searched.

S1.1 Search overview

Database	Platform	Date searched	Records
MEDLINE (PubMed)	NLM	2 July 2026	255
Embase	Elsevier	3 July 2026	383
Web of Science Core Collection (SCI-EXPANDED)	Clarivate	3 July 2026	76
Cochrane CENTRAL	Wiley	2 July 2026	51
**Total**			**765**
Duplicates removed			197
**Unique records screened**			**568**

Databases were searched from inception, with **no date or language restriction**. Scopus and SPORTDiscus were pre-specified in the protocol but could not be accessed through institutional subscriptions; this deviation is reported in the main text and was mitigated by backward and forward citation tracking of all included studies and of the key prior reviews.

S1.2 Concept blocks

The strategy combined three concept blocks with the Boolean operator **AND**; terms within each block were combined with **OR**:

- **Block 1 — Intervention**: transcranial direct current stimulation and related terms
- **Block 2 — Target**: primary motor cortex and corticospinal terms
- **Block 3 — Outcome**: jump, explosive power and force-time terms

Final combination: Block 1 AND Block 2 AND Block 3

Restrictions on population (healthy active adults), comparator (sham) and study design (randomised parallel or crossover trials) were **applied at the screening stage rather than within the search string**, in order to maximise sensitivity — standard practice in systematic review searching.

S1.3 MEDLINE (PubMed) — 2 July 2026, 255 records

```
#1  "Transcranial Direct Current Stimulation"[Mesh]
#2  ("transcranial direct current stimulation"[tiab] OR tDCS[tiab] OR
    "transcranial direct-current stimulation"[tiab] OR "anodal stimulation"[tiab] OR
    anodal[tiab] OR "brain stimulation"[tiab] OR neuromodulation[tiab] OR
    "transcranial electrical stimulation"[tiab] OR tES[tiab] OR
    "direct current stimulation"[tiab])
#3  #1 OR #2

#4  "Motor Cortex"[Mesh]
#5  "Pyramidal Tracts"[Mesh]
#6  ("motor cortex"[tiab] OR "motor cortical"[tiab] OR M1[tiab] OR "primary motor"[tiab] OR
    "primary motor cortex"[tiab] OR corticospinal[tiab] OR corticomotor[tiab] OR
    "cortico-spinal"[tiab])
#7  #4 OR #5 OR #6
```

#8 "Muscle Strength"[Mesh]
#9 "Athletic Performance"[Mesh]
#10 "Exercise"[Mesh]
#11 "Plyometric Exercise"[Mesh]
#12 (jump[tiab] OR jumps[tiab] OR jumping[tiab] OR CMJ[tiab] OR CMJA[tiab] OR countermovement[tiab] OR "counter-movement"[tiab] OR "squat jump"[tiab] OR "drop jump"[tiab] OR "vertical jump"[tiab] OR "vertical-jump"[tiab] OR plyometric[tiab] OR plyometrics[tiab] OR explosive[tiab] OR "explosive power"[tiab] OR "explosive strength"[tiab] OR ballistic[tiab] OR "muscle power"[tiab] OR "muscular power"[tiab] OR "lower-limb power"[tiab] OR "lower limb power"[tiab] OR "peak power"[tiab] OR "mean power"[tiab] OR "rate of force development"[tiab] OR RFD[tiab] OR "peak force"[tiab] OR "force-time"[tiab] OR "force time"[tiab])
#13 #8 OR #9 OR #10 OR #11 OR #12

#14 #3 AND #7 AND #13 ← executed

S1.4 Embase (embase.com) — 3 July 2026, 383 records

#1 'transcranial direct current stimulation'/exp
#2 ('transcranial direct current stimulation':ab,ti OR 'tdcs':ab,ti OR 'transcranial direct-current stimulation':ab,ti OR 'anodal stimulation':ab,ti OR 'anodal':ab,ti OR 'brain stimulation':ab,ti OR 'neuromodulation':ab,ti OR 'transcranial electrical stimulation':ab,ti OR 'tes':ab,ti OR 'direct current stimulation':ab,ti)
#3 #1 OR #2

#4 'motor cortex'/exp
#5 'pyramidal tract'/exp
#6 ('motor cortex':ab,ti OR 'motor cortical':ab,ti OR 'm1':ab,ti OR 'primary motor':ab,ti OR 'primary motor cortex':ab,ti OR 'corticospinal':ab,ti OR 'corticomotor':ab,ti OR 'cortico-spinal':ab,ti)
#7 #4 OR #5 OR #6

#8 'muscle strength'/exp
#9 'athletic performance'/exp
#10 'plyometrics'/exp
#11 ('jump':ab,ti OR 'jumps':ab,ti OR 'jumping':ab,ti OR 'cmj':ab,ti OR 'cmja':ab,ti OR 'countermovement':ab,ti OR 'counter-movement':ab,ti OR 'squat jump':ab,ti OR 'drop jump':ab,ti OR 'vertical jump':ab,ti OR 'plyometric':ab,ti OR 'plyometrics':ab,ti OR 'explosive':ab,ti OR 'explosive power':ab,ti OR 'explosive strength':ab,ti OR 'ballistic':ab,ti OR 'muscle power':ab,ti OR 'muscular power':ab,ti OR 'lower limb power':ab,ti OR 'lower-limb power':ab,ti OR 'peak power':ab,ti OR 'mean power':ab,ti OR 'rate of force development':ab,ti OR 'rfd':ab,ti OR 'peak force':ab,ti OR 'force-time':ab,ti OR 'force time':ab,ti)
#12 #8 OR #9 OR #10 OR #11

#13 #3 AND #7 AND #12 ← executed

S1.5 Web of Science Core Collection (SCI-EXPANDED) — 3 July 2026, 76 records

TS=("transcranial direct current stimulation" OR tDCS OR "anodal stimulation" OR anodal OR "brain stimulation" OR neuromodulation OR "transcranial electrical stimulation" OR tES OR "direct current stimulation")
AND
TS=("motor cortex" OR "motor cortical" OR M1 OR "primary motor" OR corticospinal OR corticomotor)
AND
TS=(jump OR jumps OR jumping OR CMJ OR CMJA OR countermovement OR "squat jump" OR "drop jump" OR "vertical jump" OR plyometric OR plyometrics OR explosive OR "explosive power" OR ballistic OR "muscle power" OR "muscular power" OR "lower limb power" OR "peak power" OR "mean power" OR "rate of force development" OR

RFD OR "peak force" OR "force-time")

S1.6 Cochrane CENTRAL (Wiley, Search Manager) — 2 July 2026, 51 records

```
#1   MeSH descriptor: [Transcranial Direct Current Stimulation] explode all trees
#2   ("transcranial direct current stimulation" OR tDCS OR "anodal stimulation" OR anodal OR
     "brain stimulation" OR neuromodulation OR "transcranial electrical stimulation" OR
     tES OR "direct current stimulation"):ti,ab,kw
#3   #1 OR #2

#4   MeSH descriptor: [Motor Cortex] explode all trees
#5   MeSH descriptor: [Pyramidal Tracts] explode all trees
#6   ("motor cortex" OR "motor cortical" OR M1 OR "primary motor" OR corticospinal OR
     corticomotor OR "cortico-spinal"):ti,ab,kw
#7   #4 OR #5 OR #6

#8   MeSH descriptor: [Muscle Strength] explode all trees
#9   MeSH descriptor: [Athletic Performance] explode all trees
#10  MeSH descriptor: [Plyometric Exercise] explode all trees
#11  (jump OR jumps OR jumping OR CMJ OR CMJA OR countermovement OR "squat jump" OR
     "drop jump" OR "vertical jump" OR plyometric OR plyometrics OR explosive OR
     "explosive power" OR ballistic OR "muscle power" OR "muscular power" OR
     "lower limb power" OR "peak power" OR "mean power" OR
     "rate of force development" OR RFD OR "peak force" OR "force-time"):ti,ab,kw
#12  #8 OR #9 OR #10 OR #11

#13  #3 AND #7 AND #12        ← executed; results restricted to the "Trials" tab (CENTRAL)
```

S1.7 Supplementary searching

Backward and forward citation tracking was performed on all included studies and on the principal prior reviews in this field (Machado et al. 2019; Holgado et al. 2019; Alix-Fages et al. 2019; Winker et al. 2024; Maudrich et al. 2022; Chinzara et al. 2022; and the umbrella review of Holgado et al. 2024). Reference lists of these reviews were hand-searched.
