# Supplementary material for: Anodal Motor-Cortex Transcranial Direct Current Stimulation and Explosive Lower-Limb Power in Healthy Active Adults: A Sham-Controlled Systematic Review and Meta-Analysis
Source: Sports (Basel). 2026 Sep 11;14(9):401. doi: 10.3390/sports14090401 (PMC13611768; doi:10.3390/sports14090401)
Supplement: Supplementary file 1 [file sports-14-00401-s001.zip › Table_S2_PRISMA_2020_Checklist.pdf]

Table S2. PRISMA 2020 Checklist

Systematic review: Anodal Motor-Cortex Transcranial Direct Current Stimulation and Explosive Lower-Limb Power in Healthy Active Adults: A Sham-Controlled Systematic Review and Meta-Analysis. Locations refer to the numbered lines of the revised manuscript.

Section and Topic	Item #	Checklist item	Location where item is reported
**TITLE**			
Title	1	Identify the report as a systematic review.	Title (lines 3–4)
**ABSTRACT**			
Abstract	2	See the PRISMA 2020 for Abstracts checklist.	Abstract (lines 9–23)
**INTRODUCTION**			
Rationale	3	Describe the rationale for the review in the context of existing knowledge.	Section 1 (lines 26–59)
Objectives	4	Provide an explicit statement of the objective(s) or question(s) the review addresses.	Section 1 (lines 26–59)
**METHODS**			
Eligibility criteria	5	Specify the inclusion and exclusion criteria for the review and how studies were grouped for the syntheses.	Section 2.2 (lines 65–78); Table 1 (lines 75–78)
Information sources	6	Specify all databases, registers, websites, organisations, reference lists and other sources searched or consulted, with the date each was last searched.	Section 2.3 (lines 79–100); Table S1
Search strategy	7	Present the full search strategies for all databases, registers and websites, including any filters and limits used.	Section 2.3 (lines 79–100); Table S1
Selection process	8	Specify the methods used to decide whether a study met the inclusion criteria, including how many reviewers screened each record and each report, whether they worked independently, and if applicable, details of automation tools used.	Section 2.4 (lines 101–137)
Data collection process	9	Specify the methods used to collect data from reports, including how many reviewers collected data from each report, whether they worked independently, any processes for obtaining or confirming data from study investigators, and if applicable, automation tools used.	Section 2.4 (lines 101–137)
Data items	10a	List and define all outcomes for which data were sought. Specify whether all results compatible with each outcome domain were sought, and if not, the methods used to decide which results to collect.	Section 2.2 (lines 65–78); Section 2.4 (lines 101–137); Table 1 (lines 75–78)
Data items	10b	List and define all other variables for which data were sought. Describe any assumptions made about any missing or unclear information.	Section 2.4 (lines 101–137); Table 2 (lines 215–218)
Study risk of bias assessment	11	Specify the methods used to assess risk of bias in the included studies, including details of the tool(s) used, how many reviewers assessed each study and whether they worked independently, and if applicable, automation tools used.	Section 2.5 (lines 138–142); Table S3
Effect measures	12	Specify for each outcome the effect measure(s) (e.g., risk ratio, mean difference) used in the synthesis or presentation of results.	Section 2.6 (lines 143–173)
Synthesis methods	13a	Describe the processes used to decide which studies were eligible for each synthesis (e.g., tabulating the study intervention characteristics and comparing against the planned groups for each synthesis).	Section 2.2 (lines 65–78); Section 2.6 (lines 143–173)
Synthesis methods	13b	Describe any methods required to prepare the data for presentation or synthesis, such as handling of missing summary statistics, or data conversions.	Section 2.4 (lines 101–137); Section 2.6 (lines 143–173)
Synthesis methods	13c	Describe any methods used to tabulate or visually display results of individual studies and syntheses.	Section 2.6 (lines 143–173); Figure 3 (lines 309–317); Figure 4 (lines 332–338)
Synthesis methods	13d	Describe any methods used to synthesise results and provide a rationale for the choice(s). If meta-analysis was performed, describe the model(s), method(s) to identify the presence and extent of statistical heterogeneity, and software package(s) used.	Section 2.6 (lines 143–173)

Section and Topic	Item #	Checklist item	Location where item is reported
Synthesis methods	13e	Describe any methods used to explore possible causes of heterogeneity among study results (e.g., subgroup analysis, meta-regression).	Section 2.6 (lines 143–173); Section 4.4 (lines 461–481)
Synthesis methods	13f	Describe any sensitivity analyses conducted to assess robustness of the synthesised results.	Section 2.6 (lines 143–173)
Reporting bias assessment	14	Describe any methods used to assess risk of bias due to missing results in a synthesis (arising from reporting biases).	Section 2.6 (lines 143–173); Section 3.5 (lines 339–355)
Certainty assessment	15	Describe any methods used to assess certainty (or confidence) in the body of evidence for an outcome.	Section 2.5 (lines 138–142); Table 5 (lines 352–355); Table S4
**RESULTS**			
Study selection	16a	Describe the results of the search and selection process, from the number of records identified in the search to the number of studies included in the review, ideally using a flow diagram.	Section 3.1 (lines 175–197); Figure 1 (line 197)
Study selection	16b	Cite studies that might appear to meet the inclusion criteria, but which were excluded, and explain why they were excluded.	Section 3.1 (lines 175–197); Section 4.5 (lines 482–520; one report excluded after assessment [41] and three not retrieved [38–40]); Figure 1 (line 197)
Study characteristics	17	Cite each included study and present its characteristics.	Section 3.2 (lines 198–221); Table 2 (lines 215–218); Table 3 (lines 254–258); Table 4 (lines 277–280)
Risk of bias in studies	18	Present assessments of risk of bias for each included study.	Section 3.2 (lines 198–221); Figure 2 (lines 220–221); Table S3
Results of individual studies	19	For all outcomes, present, for each study: (a) summary statistics for each group (where appropriate) and (b) an effect estimate and its precision (e.g., confidence/credible interval), ideally using structured tables or plots.	Figure 3 (lines 309–317); Figure 4 (lines 332–338)
Results of syntheses	20a	For each synthesis, briefly summarise the characteristics and risk of bias among contributing studies.	Section 3.3 (lines 281–317); Section 3.4 (lines 318–338); Section 3.5 (lines 339–355)
Results of syntheses	20b	Present results of all statistical syntheses conducted. If meta-analysis was done, present for each the summary estimate and its precision (e.g., confidence/credible interval) and measures of statistical heterogeneity. If comparing groups, describe the direction of the effect.	Section 3.3 (lines 281–317); Section 3.4 (lines 318–338); Figure 3 (lines 309–317); Figure 4 (lines 332–338)
Results of syntheses	20c	Present results of all investigations of possible causes of heterogeneity among study results.	Section 3.3 (lines 281–317); Section 4.4 (lines 461–481)
Results of syntheses	20d	Present results of all sensitivity analyses conducted to assess the robustness of the synthesised results.	Section 3.3 (lines 281–317); Section 3.4 (lines 318–338); Section 3.6 (lines 356–364)
Reporting biases	21	Present assessments of risk of bias due to missing results (arising from reporting biases) for each synthesis assessed.	Section 3.5 (lines 339–355); not formally performed ($k < 10$)
Certainty of evidence	22	Present assessments of certainty (or confidence) in the body of evidence for each outcome assessed.	Section 3.5 (lines 339–355); Table 5 (lines 352–355); Table S4
**DISCUSSION**			
Discussion	23a	Provide a general interpretation of the results in the context of other evidence.	Section 4.1 (lines 366–386); Section 4.2 (lines 387–418)
Discussion	23b	Discuss any limitations of the evidence included in the review.	Section 4.5 (lines 482–520)

Section and Topic	Item #	Checklist item	Location where item is reported
Discussion	23c	Discuss any limitations of the review processes used.	Section 4.5 (lines 482–520)
Discussion	23d	Discuss implications of the results for practice, policy, and future research.	Section 4.6 (lines 521–546); Section 4.7 (lines 547–567); Section 5 (lines 568–578)
**OTHER INFORMATION**			
Registration and protocol	24a	Provide registration information for the review, including the register name and registration number, or state that the review was not registered.	Section 2.1 (lines 61–64); PROSPERO CRD420261438841
Registration and protocol	24b	Indicate where the review protocol can be accessed, or state that a protocol was not prepared.	Section 2.1 (lines 61–64); PROSPERO CRD420261438841
Registration and protocol	24c	Describe and explain any amendments to information provided at registration or in the protocol.	Section 2.1 (lines 61–64); Section 4.5 (lines 482–520)
Support	25	Describe sources of financial or non-financial support for the review, and the role of the funders or sponsors in the review.	Funding (line 592)
Competing interests	26	Declare any competing interests of review authors.	Conflicts of Interest (line 613)
Availability of data, code and other materials	27	Report which of the following are publicly available and where they can be found: template data collection forms; data extracted from included studies; data used for all analyses; analytic code; any other materials used in the review.	Data Availability Statement (line 602); Supplementary Materials (line 579); Tables S1–S4

From: Page MJ, McKenzie JE, Bossuyt PM, et al. The PRISMA 2020 statement: an updated guideline for reporting systematic reviews. BMJ 2021;372:n71. doi:10.1136/bmj.n71. For more information, visit: http://www.prisma-statement.org/
