# Supplementary material for: Anodal Motor-Cortex Transcranial Direct Current Stimulation and Explosive Lower-Limb Power in Healthy Active Adults: A Sham-Controlled Systematic Review and Meta-Analysis
Source: Sports (Basel). 2026 Sep 11;14(9):401. doi: 10.3390/sports14090401 (PMC13611768; doi:10.3390/sports14090401)
Supplement: Supplementary file 1 [file sports-14-00401-s001.zip › Table_S3_RoB2_assessment.pdf]

Table S3. Risk-of-Bias (RoB 2) Assessments for the Seven Included Studies

Per-study, per-domain risk-of-bias judgements using the Cochrane Risk of Bias 2 (RoB 2) tool (parallel and crossover versions). Domains: D1, randomisation process; D2, deviations from intended interventions (including blinding of participants and personnel); D3, missing outcome data; D4, measurement of the outcome; D5, selection of the reported result. The overall judgement is the least favourable domain rating.

Study	D1	D2	D3	D4	D5	Overall
Lattari 2020	− Some concerns	− Some concerns	+ Low	+ Low	− Some concerns	− Some concerns
Lu 2021	− Some concerns	− Some concerns	+ Low	+ Low	− Some concerns	− Some concerns
Zhu 2023	− Some concerns	− Some concerns	− Some concerns	+ Low	− Some concerns	− Some concerns
Song 2023	− Some concerns	− Some concerns	+ Low	− Some concerns	− Some concerns	− Some concerns
Park 2023	− Some concerns	− Some concerns	+ Low	+ Low	− Some concerns	− Some concerns
Rocha 2024	+ Low	− Some concerns	+ Low	+ Low	− Some concerns	− Some concerns
Chen 2026	− Some concerns	− Some concerns	+ Low	+ Low	+ Low	− Some concerns

Summary: all seven included trials were rated "some concerns" overall. No trial was rated low risk overall and no trial was rated high risk. The recurring concerns were unverified success of sham blinding (D2), unreported allocation concealment (D1), and the absence of a prospective registration or protocol (D5). The traffic-light plot is shown as Figure 2 in the main text. Zhou et al. (2024) is not assessed here: its comparator was a no-treatment control rather than a sham, and it is therefore outside the review's eligibility criteria (see Section 3.1 of the main text).

Reference: Sterne JAC, Savović J, Page MJ, et al. RoB 2: a revised tool for assessing risk of bias in randomised trials. BMJ 2019;366:l4898. doi:10.1136/bmj.l4898.
