# Supplementary material for: Anodal Motor-Cortex Transcranial Direct Current Stimulation and Explosive Lower-Limb Power in Healthy Active Adults: A Sham-Controlled Systematic Review and Meta-Analysis
Source: Sports (Basel). 2026 Sep 11;14(9):401. doi: 10.3390/sports14090401 (PMC13611768; doi:10.3390/sports14090401)
Supplement: Supplementary file 1 [file sports-14-00401-s001.zip › Table_S4_GRADE_profile.pdf]

## Table S4. GRADE Summary of Findings

Anodal M1 tDCS versus sham for explosive lower-limb power in healthy active adults. Population: healthy, recreationally active to trained adults (18-45 years). Intervention: anodal M1 tDCS. Comparison: sham tDCS. Effect measure: standardised mean difference (Hedges' g), random-effects model (REML with Hartung-Knapp-Sidik-Jonkman interval).

| **Outcome category** | **No. of trials (k)** | **Participants** | **Pooled Hedges' g (95% CI)** | **Certainty (GRADE)** | **Downgrading reasons** |
|---|---|---|---|---|---|
| Jump output | 6 | 76 per condition | 0.44 (−0.05 to 0.92) | ⨁○○○ Very low | Risk of bias (−1)(a); indirectness (−1)(b); imprecision (−1)(c) |
| Force-time kinetics | 3 | 48 per condition | 0.67 (0.53 to 0.81) Hartung-Knapp; −0.23 to 1.57 under a conservative small-sample variance correction | ⨁○○○ Very low | Risk of bias (−1)(a); indirectness (−1)(b); imprecision (−1)(c) |

### GRADE Working Group grades of evidence

High certainty: We are very confident that the true effect lies close to that of the estimate. Moderate certainty: We are moderately confident in the effect estimate; the true effect is likely to be close, but there is a possibility that it is substantially different. Low certainty: Our confidence in the effect estimate is limited; the true effect may be substantially different. Very low certainty: We have very little confidence in the effect estimate; the true effect is likely to be substantially different from the estimate of effect.

### Explanations

(a) Risk of bias: all seven trials were rated "some concerns" overall on RoB 2; none was rated low risk and none was rated high risk. Success of sham blinding was never verified and allocation concealment was rarely reported (downgraded one level).

(b) Indirectness: outcomes are laboratory-based surrogates of athletic performance (jump height, rate of force development, peak power/force) rather than competition performance, obtained across heterogeneous montages, populations, and dosing protocols (downgraded one level).

(c) Imprecision: k = 6 and k = 3 respectively, with wide intervals. For jump output the interval includes both no effect and a large benefit and the 95% prediction interval was −0.27 to 1.14; for force-time kinetics the effect does not survive the conservative small-sample variance correction, under which the interval includes no effect. Both categories comprised fewer than ten trials (downgraded one level).

Note: For force-time kinetics the Hartung-Knapp interval (0.53 to 0.81) is shown together with the interval obtained under a conservative small-sample variance correction (−0.23 to 1.57; $p = 0.086$). With $k = 3$ and $\tau^2 = 0$ the Hartung-Knapp interval is liable to be anti-conservative — its pooled standard error, 0.032, is an order of magnitude smaller than that of any contributing trial — and the corrected interval is the one on which inference should rest.
